# Gut-derived metabolites and mitochondrial homeostasis in heart failure: mechanistic links and translational perspectives

**DOI:** 10.3389/fcvm.2026.1917012

**Published:** 2026-08-03

**Authors:** Daqiu Chen, Mingxuan Su, Zhanxiong Xie, Shanghua Xu, Shunxiang Luo

**Affiliations:** 1 Department of Cardiology, Nanping First Hospital Affiliated to Fujian Medical University, Nanping, Fujian Province, China; 2 Department of Cardiology, Nanping Second Hospital, Nanping, Fujian, China

**Keywords:** context-dependent effects, gut microbiota, gut-derived metabolites, heart failure, mitochondrial quality control, myocardial mitochondrial homeostasis, short-chain fatty acids, trimethylamine N-oxide

## Abstract

Heart failure is a chronic cardiovascular syndrome with high morbidity and mortality worldwide, and its progression is closely linked to myocardial metabolic remodeling and disruption of mitochondrial homeostasis. Increasing evidence suggests that the gut microbiota and its metabolites represent an important interface between diet, inflammation, metabolic stress, and cardiovascular remodeling. Gut-derived metabolites, including short-chain fatty acids, trimethylamine N-oxide, tryptophan-derived metabolites, bile acids, phenylacetylglutamine, indoxyl sulfate, and urolithins, may influence myocardial mitochondrial homeostasis by affecting substrate oxidation, oxidative phosphorylation, reactive oxygen species production, inflammatory signaling, mitochondrial dynamics, mitophagy, and cell-death pathways. However, these metabolites should not be interpreted as uniformly protective or detrimental, because their biological effects may depend on concentration, exposure duration, bioavailability, protein binding, renal clearance, cellular targets, host metabotype, experimental model, and heart failure phenotype. Short-chain fatty acids and indole-3-propionic acid (IPA) have been linked to mitochondrial oxidative metabolism, nicotinamide adenine dinucleotide (NAD+)/sirtuin 3 (SIRT3)-related mitochondrial signaling, and inflammatory regulation in selected experimental settings, whereas the choline/trimethylamine N-oxide axis, indoxyl sulfate, phenylacetylglutamine, and dysregulated bile acid metabolism are associated with myocardial fibrosis, oxidative stress, mitochondrial dysfunction, and adverse outcomes. Nevertheless, many clinical associations may be influenced by renal dysfunction, disease severity, and heart-to-gut reverse causality, and many mechanistic findings remain derived from animal models, *ex vivo* systems, or non-classical heart-failure models. This narrative review summarizes current evidence linking gut-derived metabolites to myocardial mitochondrial homeostasis in heart failure, with emphasis on energy metabolic remodeling, oxidative stress, inflammation, mitochondrial quality control, cell death, and fibrotic remodeling. Potential intervention strategies targeting the gut microbiota and its metabolic pathways are also discussed with attention to their translational limitations and to the need for direct mitochondrial readouts, cell-type-specific validation, and phenotype-specific clinical studies.

## 1 Introduction

Heart failure is a common clinical syndrome and remains a leading cause of morbidity and mortality worldwide. It often represents the advanced or end-stage manifestation of diverse cardiovascular diseases and involves multiple mechanisms, including neuroendocrine activation, inflammation, oxidative stress, myocardial fibrosis, and impaired energy metabolism (1, 2). Cardiomyocytes are highly dependent on mitochondria for energy supply. Beyond oxidative phosphorylation and ATP production, mitochondria also participate in calcium homeostasis, reactive oxygen species (ROS) generation, cell death, and innate immune signaling (3, 4). In the setting of heart failure, myocardial mitochondria commonly exhibit impaired biogenesis, restricted substrate oxidation, reduced respiratory chain efficiency, disturbed mitochondrial dynamics, and defective mitophagy. These abnormalities further contribute to energy deficiency, increased oxidative stress, and aggravated cellular injury (5, 6). Therefore, the maintenance of mitochondrial homeostasis is regarded as an important mechanistic basis for delaying myocardial remodeling and the deterioration of cardiac function.

**Figure 1:**
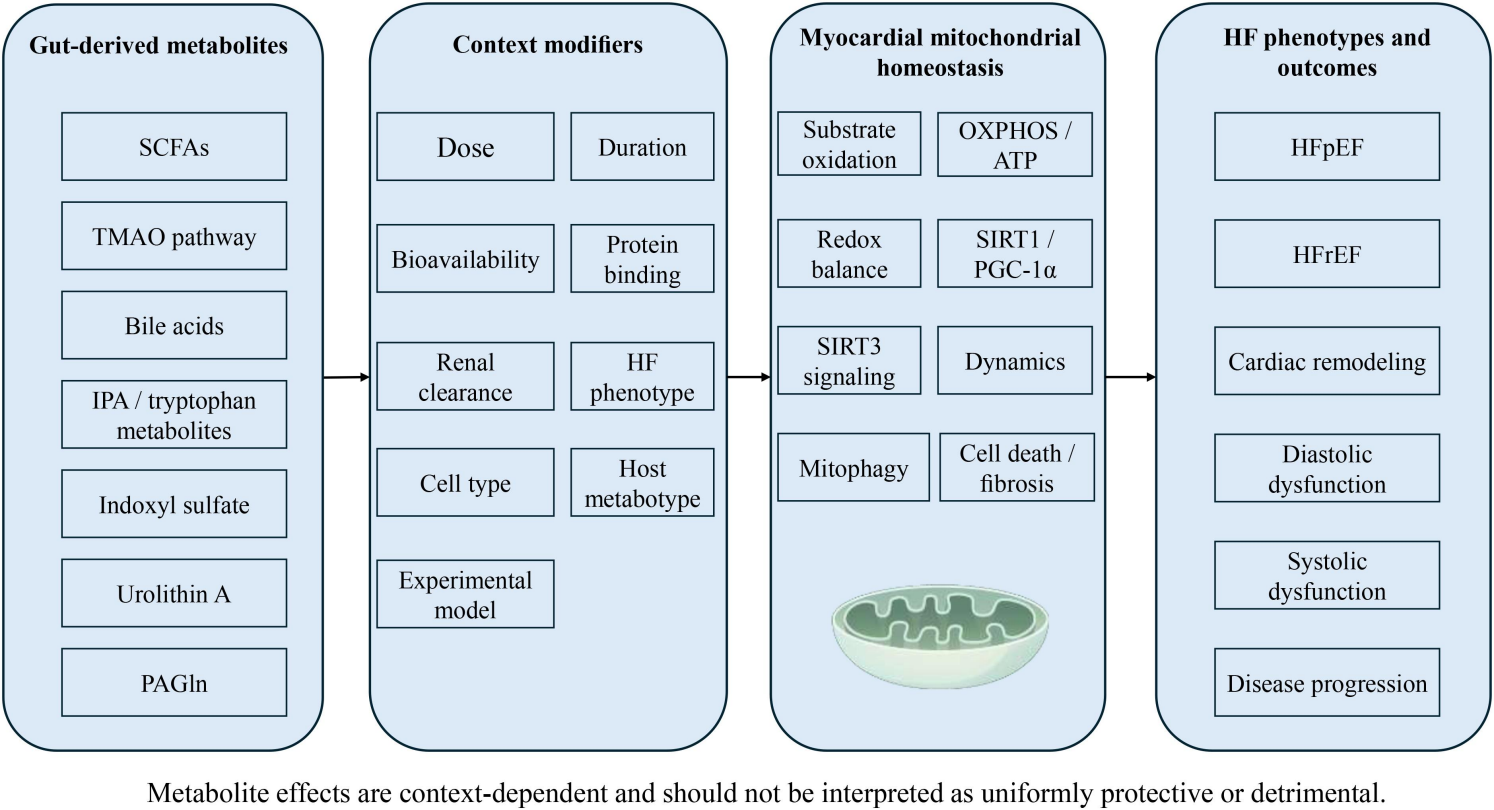
Context-dependent gut-derived metabolite effects on myocardial mitochondrial homeostasis in heart failure. Gut-derived metabolites may influence mitochondrial substrate oxidation, oxidative phosphorylation, ATP production, redox balance, SIRT1/PGC-1*α*-related mitochondrial biogenesis, SIRT3-related antioxidant and metabolic regulation, mitochondrial dynamics, mitophagy, cell death, and fibrotic remodeling. These effects may be modified by metabolite concentration, exposure duration, bioavailability, protein binding, renal clearance, cell type, heart failure phenotype, host metabotype, and experimental model, and may contribute to HFpEF, HFrEF, cardiac remodeling, diastolic dysfunction, systolic dysfunction, and disease progression. This framework emphasizes context-dependent effects rather than a simple directional classification. ATP, adenosine triphosphate; HF, heart failure; HFpEF, heart failure with preserved ejection fraction; HFrEF, heart failure with reduced ejection fraction; IPA, indole-3-propionic acid; OXPHOS, oxidative phosphorylation; PAGln, phenylacetylglutamine; PGC-1*α*, peroxisome proliferator-activated receptor gamma coactivator 1-alpha; SCFAs, short-chain fatty acids; SIRT1, sirtuin 1; SIRT3, sirtuin 3; TMAO, trimethylamine N-oxide.

In recent years, the role of the gut microbiota and its metabolites in cardiovascular diseases has received increasing attention. Patients with heart failure frequently exhibit gut microbial dysbiosis, altered microbial metabolic capacity, impaired intestinal barrier function, and enhanced systemic low-grade inflammation (7–11). Importantly, these gut abnormalities may not only contribute to heart failure progression but may also arise as a consequence of heart failure itself, because reduced intestinal perfusion, venous congestion, bowel wall edema, and barrier dysfunction can reshape the gut microbial ecosystem and circulating metabolite profiles. Gut-derived metabolites may reach the heart through the circulation and may also affect cardiac cellular function through immune-inflammatory regulation, metabolic substrate supply, receptor-mediated signaling, and epigenetic modulation (8, 12–14). However, their biological effects should not be interpreted as uniformly protective or detrimental. Instead, the direction and magnitude of their effects may depend on metabolite concentration, exposure duration, bioavailability, protein binding, renal clearance, cellular target, heart failure phenotype, and experimental model. Notably, gut-derived metabolites do not exert uniform effects. Short-chain fatty acids, indole-3-propionic acid, and urolithin A have been linked to mitochondrial metabolic support or quality-control pathways in selected experimental settings, whereas trimethylamine N-oxide, indoxyl sulfate, phenylacetylglutamine, and certain abnormalities in bile acid metabolism have been associated with adverse remodeling, mitochondrial dysfunction, or worse clinical outcomes in specific disease contexts. Therefore, this review focuses on the context-dependent axis of “gut-derived metabolites–myocardial mitochondrial homeostasis–heart failure phenotypes,” while emphasizing the limitations related to causality, renal confounding, metabolite exposure, and cell-type specificity. This framework may help integrate currently fragmented basic and clinical evidence and provide new research directions for mechanistically informed and phenotype-specific investigation in heart failure. A context-dependent conceptual framework linking gut-derived metabolites, myocardial mitochondrial homeostasis, and heart failure phenotypes and outcomes is summarized in Figure 1.

### 1.1 Literature search strategy and scope

This article is a narrative review rather than a systematic review or meta-analysis. Relevant studies were identified through searches of PubMed, Web of Science, Embase, and Google Scholar, with emphasis on publications related to heart failure, gut microbiota, gut-derived metabolites, short-chain fatty acids, trimethylamine N-oxide, tryptophan-derived metabolites, bile acids, urolithins, myocardial mitochondria, mitochondrial homeostasis, mitophagy, mitochondrial dynamics, oxidative stress, inflammation, and fibrosis. Priority was given to mechanistic studies, translational investigations, clinical cohort studies, randomized trials, and high-quality reviews published in recent years. Foundational studies were also included when they provided essential mechanistic or conceptual context. Because this review aimed to synthesize mechanistic links and translational perspectives, formal study selection, risk-of-bias assessment, and quantitative evidence synthesis were not performed.

## 2 Major gut-derived metabolites and their context-dependent effects on myocardial mitochondria

### 2.1 Regulation of myocardial mitochondrial energy metabolism by short-chain fatty acids

Short-chain fatty acids (SCFAs), mainly including acetate, propionate, and butyrate, are major products of dietary fiber fermentation by the gut microbiota. They exert broad biological effects through activation of G protein-coupled receptors, such as GPR41 and GPR43, and inhibition of histone deacetylases (HDACs) (15). In myocardial energy metabolism, SCFAs may function either as supplementary metabolic substrates or as signaling molecules. Acetate can be converted into acetyl-CoA by acetyl-CoA synthetase, butyrate can enter the tricarboxylic acid cycle through fatty acid oxidation, whereas propionate can be converted into propionyl-CoA and further participate in succinyl-CoA-related metabolic pathways. Therefore, different SCFAs may exert substrate-specific effects on myocardial energy metabolism.

Notably, in pressure overload-induced failing hearts, SCFAs can be oxidized by the myocardium, and their oxidation rate exceeds that of ketone bodies, suggesting that failing hearts retain the capacity to oxidize SCFAs under experimental conditions rather than proving that circulating SCFAs provide quantitatively important fuel in human heart failure (16). Butyrate has been reported to possess anti-inflammatory, antioxidant, and mitochondria-protective potential in studies of metabolic diseases and cardiac injury (17, 18). However, its direct regulatory effects on mitochondrial permeability transition pore (mPTP) opening and cardiomyocyte apoptosis require further validation in heart-specific experimental settings. In addition, propionate and other SCFAs may participate in metabolic and inflammatory regulation through pathways such as GPR41/GPR43 (19), but their direct roles in myocardial mitophagy remain to be further clarified. Overall, SCFAs may influence myocardial energy metabolism through metabolic substrate supply, receptor-mediated signaling, and epigenetic regulation; however, their effects may depend on metabolite subtype, concentration, heart failure phenotype, and disease stage.

### 2.2 Context-dependent effects of trimethylamine N-oxide on cardiac function and mitochondrial metabolism

Trimethylamine N-oxide (TMAO) is a gut microbiota-derived metabolite generated from trimethylamine (TMA), which is produced through microbial metabolism of dietary choline and L-carnitine and subsequently oxidized by hepatic flavin-containing monooxygenase 3 (FMO3) (20). Epidemiological studies have shown that elevated plasma TMAO levels are closely associated with adverse outcomes in patients with heart failure (21, 22). At the molecular level, studies in cardiac injury models have demonstrated that TMAO can exacerbate myocardial ischemia–reperfusion injury and is associated with mitochondrial dysfunction, enhanced oxidative stress, and sustained activation of endoplasmic reticulum stress (23). In other vascular injury models, TMAO-related inflammasome activation and oxidative stress have also been observed (24), suggesting that TMAO may indirectly promote cardiovascular remodeling through an inflammation–oxidative stress network.

Importantly, not all experimental findings support a uniformly detrimental role of TMAO. Oakley et al. reported that acute exposure to TMAO increased cardiac muscle contractility, suggesting that the biological effects of TMAO may depend on exposure duration, concentration, experimental setting, and disease context (25). Therefore, TMAO should be interpreted as a context-dependent gut-derived metabolite rather than as a uniformly harmful molecule. Although chronically elevated TMAO levels are consistently associated with adverse outcomes in patients with heart failure, its causal role, myocardial accessibility, and precise mitochondrial mechanisms still require further validation.

### 2.3 Bile acid dysregulation, myocardial metabolic disturbance, and heart failure progression

Bile acids are not only involved in lipid digestion but also function as signaling molecules within the gut microbiota–liver–heart axis. Patients with chronic heart failure exhibit altered bile acid profiles, characterized by reduced primary bile acids and increased levels of specific secondary bile acids, and these alterations are associated with poorer survival (26). Animal studies have shown that bile acid overload can reduce fatty acid oxidation in cardiomyocytes and induce myocardial hypertrophy, bradycardia, and impaired exercise tolerance, suggesting a potential link between bile acid dysregulation and cardiac dysfunction in selected experimental settings (27).

Heart failure progression is frequently accompanied by disturbances in mitochondrial fission, fusion, and mitophagy. Among these processes, DRP1-mediated excessive mitochondrial fission is considered to be closely associated with myocardial energy failure, oxidative stress, and cell death (28). Therefore, bile acid dysregulation may participate in heart failure progression by affecting myocardial energy metabolism, oxidative stress, and mitochondrial function. However, whether bile acids directly regulate mitochondrial dynamics proteins, such as DRP1, MFN1/2, or OPA1, requires further validation in heart failure-specific experimental models.

### 2.4 Tryptophan-related metabolites and regulation of myocardial mitochondrial homeostasis

Tryptophan can be metabolized through the kynurenine, melatonin, and gut microbiota-derived indole pathways, and these metabolites are closely associated with cardiovascular disease and heart failure progression (29, 30). Myocardial mitophagy is an essential mechanism for maintaining mitochondrial quality control and energy homeostasis. Dysregulated mitophagy can promote oxidative stress, cardiomyocyte death, and heart failure progression (31).

Among tryptophan-related metabolites, melatonin has been extensively investigated for its cardioprotective effects. Studies have shown that melatonin can regulate mitophagy through the Apelin/SIRT3 axis and attenuate myocardial ischemia–reperfusion injury (32). The gut microbiota-derived metabolite indole-3-propionic acid (IPA) can improve mitochondrial respiration in cardiomyocytes and enhance cardiac function, suggesting that it may indirectly influence mitophagy by maintaining mitochondrial homeostasis (33). In contrast, excessive activation of the kynurenine pathway is associated with adverse prognosis in heart failure and pathological cardiac hypertrophy, potentially disrupting the balance of mitophagy through inflammation and mitochondrial injury (34). Therefore, tryptophan-related metabolites may participate in heart failure-associated myocardial injury by modulating mitochondrial respiration, oxidative stress, and mitochondrial quality control. However, direct evidence regarding their regulation of mitophagy in the failing myocardium remains insufficient and requires further investigation. The major gut-derived metabolites discussed in this review and their context-dependent mitochondrial relevance in heart failure are summarized in Table 1.

**Table 1:** Major gut-derived metabolites and context-dependent mitochondrial relevance in heart failure.

Metabolite/pathway	Main source or precursor	Representative HF-related evidence	Putative mitochondrial relevance	Key translational caveat	Current interpretation	Key references
SCFAs	Dietary fiber fermentation by gut microbiota; acetate, propionate, and butyrate.	*Ex vivo* failing-heart studies show SCFA oxidation; high-fiber or acetate interventions improve remodeling in selected models.	Alternative substrate oxidation and AMPK-related metabolic signaling may influence mitochondrial energy balance.	Peripheral exposure is limited by first-pass handling and bioavailability; quantitative myocardial fuel contribution in human HF remains uncertain.	Context-dependent candidate mediator/biomarker; not yet validated as a therapeutic target.	(15, 16, 35, 70, 75, 76, 79)
TMAO pathway	Dietary choline, phosphatidylcholine, and carnitine → microbial TMA → hepatic FMO-dependent TMAO.	Clinical HF cohorts and meta-analysis link TMAO to severity and outcomes; pressure-overload and HFpEF models support pathway relevance.	Experimental studies report energetic and remodeling effects, including complex IV, ATP/phosphocreatine, inflammation, and fibrosis-related readouts.	Renal clearance, exposure duration, diet, and acute vs. chronic effects complicate interpretation; not an established clinical target.	Context-dependent candidate mediator and biomarker; causality remains incompletely resolved.	(21, 22, 44, 57, 61, 69, 80–82)
Bile acid dysregulation	Gut microbiota-liver metabolism of primary and secondary bile acids.	Altered secondary/primary bile acid ratio reported in chronic HF; bile acid excess induces cardiometabolic dysfunction in models.	May regulate lipid metabolism, receptor signaling, inflammation, and metabolic remodeling.	Effects are subtype-, receptor-, hepatic-, and disease-context specific; direct HF mitochondrial evidence remains limited.	Hypothesis-generating disease-context signal; not a validated mitochondrial target.	(26, 27, 50, 51)
Tryptophan-derived metabolites/IPA	Microbial tryptophan metabolism.	IPA is reduced in an HFpEF model; supplementation improves diastolic dysfunction, oxidative stress, inflammation, and metabolic abnormalities.	Supports mitochondrial respiration, NAD+ salvage, SIRT3-related regulation, and redox/inflammatory modulation.	Evidence is mechanistically strong but mainly preclinical; dose, exposure, delivery, and human HF validation remain uncertain.	Mechanistically promising but mainly preclinical.	(33, 56, 60)
Kynurenine-pathway metabolites	Host and microbial tryptophan metabolism with inflammation-related pathway activation.	Plasma kynurenines are associated with prognosis in HF.	May reflect inflammatory-metabolic activation rather than direct myocardial mitochondrial regulation.	Renal function, immune activation, and systemic disease burden may confound circulating levels.	Disease-context biomarker; hypothesis-generating signal.	(30, 34)
Indoxyl sulfate	Gut microbial indole production followed by hepatic sulfation; protein-bound uremic toxin.	CKD-HF mechanistic evidence links indoxyl sulfate to HF progression and cardiomyocyte apoptosis.	Reported to impair mitochondrial function through AHR-CYP1B1-related mechanisms in cardiorenal settings.	Albumin binding, free fraction, renal clearance, and CKD context are central; extrapolation to non-CKD HF should be cautious.	Cardiorenal disease-context mediator/biomarker; not broadly generalizable.	(36–38, 40)
Urolithin A	Microbial metabolism of ellagitannins.	Preclinical cardiac injury and fibrosis models support mitochondrial quality-control effects.	May enhance mitophagy and mitochondrial quality control and reduce apoptosis/fibrosis in models.	Host metabotype and precursor conversion strongly affect exposure; human HF-specific evidence is limited.	Mechanistically promising but not clinically established in HF.	(39, 65, 66, 72)
PAGln	Microbial phenylalanine metabolism.	Associated with HF presence, disease severity, cardiovascular events, and prognosis.	Direct myocardial mitochondrial mechanisms are insufficiently established.	Renal-metabolic confounding and reverse causality limit interpretation.	Clinical risk biomarker; not yet validated as a mitochondrial therapeutic target.	(40–43)

AHR, aryl hydrocarbon receptor; AMPK, AMP-activated protein kinase; ATP, adenosine triphosphate; CKD, chronic kidney disease; CYP1B1, cytochrome P450 family 1 subfamily B member 1; FMO, flavin-containing monooxygenase; HF, heart failure; HFpEF, heart failure with preserved ejection fraction; IPA, indole-3-propionic acid; NAD+, nicotinamide adenine dinucleotide; PAGln, phenylacetylglutamine; SCFAs, short-chain fatty acids; SIRT3, sirtuin 3; TMA, trimethylamine; TMAO, trimethylamine N-oxide.

## 3 Translational constraints in interpreting gut-derived metabolite–mitochondria interactions

Before discussing specific mitochondrial signaling pathways, several translational constraints should be considered. Gut-derived metabolites are often discussed as if their circulating levels directly reflect myocardial exposure and mitochondrial activity. However, their biological effects are strongly influenced by concentration, exposure duration, bioavailability, protein binding, renal clearance, cell type, heart failure phenotype, host metabotype, and experimental model. These factors are particularly important when extrapolating findings from *in vitro* systems, *ex vivo* perfused hearts, animal models, or non-classical heart failure models to human heart failure. A detailed summary of exposure-related, bioavailability-related, renal, and evidence-level constraints for major gut-derived metabolites and community-level gut dysbiosis is provided in Supplementary Table S1.

### 3.1 Concentration, bioavailability, and myocardial exposure

A major limitation in this field is that circulating metabolite concentrations do not necessarily reflect myocardial exposure or intracellular mitochondrial concentrations. For SCFAs, intestinal luminal concentrations are much higher than systemic circulating concentrations, and molar ratios change substantially from colonic contents to portal, hepatic, and peripheral venous blood, reflecting extensive colonic epithelial uptake and hepatic first-pass handling (35). Therefore, *ex vivo* evidence showing that butyrate and other SCFAs can be oxidized by failing hearts should be interpreted as evidence of metabolic capacity rather than definitive proof that dietary or microbiota-derived SCFAs reach the failing human myocardium at sufficient concentrations to serve as quantitatively important alternative fuels (16, 35).

Protein binding and free metabolite fraction are also critical. Indoxyl sulfate is highly albumin-bound, and its biologically active free fraction may be much lower than the total plasma concentration (36, 37). *In vitro* studies that apply total indoxyl sulfate concentrations without accounting for albumin binding may therefore overestimate direct cardiomyocyte toxicity. This issue is directly relevant to interpretation of AHR–CYP1B1-mediated apoptosis and mitochondrial dysfunction in CKD-associated heart failure (38). Similarly, for TMAO, proposed mechanisms involving mitochondrial respiratory-chain impairment should be interpreted with caution unless intracellular myocardial accumulation and mitochondrial accessibility are directly demonstrated. Overall, future studies should report both physiologically relevant concentrations and experimental exposure levels, and should distinguish total circulating levels from free, bioavailable, and myocardial concentrations.

Urolithin A provides another example of a translational barrier related to host metabotype. Urolithin A is generated from ellagitannins through gut microbial metabolism, but individuals differ substantially in their ability to produce urolithins, giving rise to distinct urolithin metabotypes, including low-producer or non-producer phenotypes (39). Therefore, dietary strategies intended to increase urolithin A exposure may not produce uniform systemic or myocardial effects across patients with heart failure, and direct supplementation may differ biologically from food-based precursor strategies.

### 3.2 Renal clearance, cardiorenal confounding, and reverse causality

Renal function is a central confounder in clinical studies of gut-derived metabolites and heart failure. TMAO is strongly influenced by renal clearance, whereas indoxyl sulfate and PAGln are closely related to renal dysfunction and uremic solute biology (38, 40). Because chronic kidney disease is common in patients with heart failure, associations between these metabolites and adverse outcomes may partly reflect impaired renal clearance rather than direct cardiac toxicity. For example, PAGln has been associated with heart failure risk and prognosis, but it is also closely correlated with renal-function markers, including creatinine, blood urea nitrogen, estimated glomerular filtration rate, and cystatin C (41–43). Therefore, observational associations involving TMAO, indoxyl sulfate, and PAGln should be interpreted in light of whether renal function was measured, adjusted for, or stratified in the original studies.

Causal inference is also complicated by reverse causality. Mendelian randomization evidence has suggested that genetically predicted TMAO is not consistently associated with major cardiometabolic diseases, whereas type 2 diabetes and kidney disease may increase circulating TMAO levels, supporting the possibility that some observational associations are partly attributable to confounding or reverse causality (44). Moreover, heart failure itself can promote splanchnic hypoperfusion, venous congestion, bowel wall edema, intestinal barrier dysfunction, endotoxin translocation, and gut microbial dysbiosis. Foundational studies by Sandek, Anker, and colleagues demonstrated altered intestinal morphology and permeability in chronic heart failure, and later showed that intestinal blood flow is reduced in patients with heart failure and is linked to bacterial growth, gastrointestinal symptoms, and cachexia (45, 46). Thus, altered gut microbiota and circulating metabolites in patients with heart failure may be consequences of the failing heart as well as potential contributors to disease progression. Clinical associations are therefore compatible with both gut-to-heart and heart-to-gut pathways. This distinction is essential when interpreting TMAO- and PAGln-related prognostic associations, because biomarker associations do not by themselves establish causal involvement in myocardial mitochondrial injury.

### 3.3 Cell-type specificity and direct versus inferred mitochondrial evidence

Another unresolved issue is whose mitochondria are being affected. Myocardial tissue contains cardiomyocytes, fibroblasts, endothelial cells, vascular cells, macrophages, and resident immune cells. Some gut-derived metabolites or their receptors may act predominantly on non-cardiomyocyte populations. For example, single-cell mapping of SCFA receptors in human and mouse hearts provides a useful framework for interpreting receptor-mediated SCFA effects across cardiac cell populations rather than assuming cardiomyocyte-autonomous signaling (15). Similarly, mitochondrial dynamics in non-cardiomyocyte populations, such as macrophages, may influence post-injury cardiac inflammation and remodeling (47). Therefore, apparent effects on myocardial mitochondrial homeostasis may be mediated indirectly through inflammation, endothelial function, fibroblast activation, immune-cell metabolism, or paracrine signaling rather than through direct cardiomyocyte-autonomous mitochondrial regulation.

It is also important to distinguish direct mitochondrial measurements from inferred mitochondrial relevance. Direct evidence includes respirometry, mitochondrial complex activity, ATP or phosphocreatine measurements, mitochondrial membrane potential, mitochondrial ROS, mitochondrial calcium handling, and mitochondrial morphology. In contrast, changes in markers such as PGC-1*α*, SIRT3, PINK1/Parkin, LC3, apoptosis markers, fibrosis markers, or inflammatory mediators provide indirect or supportive evidence, but they do not by themselves prove direct mitochondrial functional regulation. Future studies should combine cell-type-specific models, single-cell or spatial omics, and direct mitochondrial functional assays to determine whether gut-derived metabolites act directly on cardiomyocyte mitochondria or indirectly through non-cardiomyocyte compartments.

## 4 Mitochondrial mechanisms linking gut-derived metabolites to heart failure: direct evidence and inferred pathways

After considering the translational constraints described above, the following section summarizes mitochondrial pathways that have been linked to gut-derived metabolites in heart failure-related settings. These pathways should be interpreted according to the strength of evidence. Some studies directly measured mitochondrial function, including substrate oxidation, respiratory capacity, mitochondrial complex activity, ATP or phosphocreatine levels, mitochondrial ROS, or mitochondrial morphology. Other studies inferred mitochondrial relevance from signaling markers, apoptosis, fibrosis, inflammatory activation, or clinical associations. Therefore, the mechanisms discussed below should be viewed as evidence-supported hypotheses rather than uniformly established causal pathways in human heart failure.

### 4.1 AMPK-related energy sensing and mitochondrial biogenesis: proposed links

Gut-derived metabolites have been proposed to influence AMPK-related energy-sensing pathways, but direct heart-failure-specific evidence remains limited. SCFAs, particularly butyrate, have been reported to modulate AMPK-related signaling in studies of heart failure and metabolic diseases. These effects may be associated with alterations in cellular energy status, receptor-mediated signaling, and HDAC inhibition (48). In contrast, TMAO may impair myocardial energy metabolism by promoting inflammatory responses, oxidative stress, and mitochondrial dysfunction (23). Some studies suggest that TMAO may participate in cardiovascular injury by affecting AMPK/PGC-1*α*-related energy metabolic signaling; however, whether TMAO directly suppresses mitochondrial biogenesis through this axis in the failing myocardium remains insufficiently supported by heart failure-specific experimental evidence (49). In addition, bile acid–TGR5/FXR signaling can regulate systemic glucose and lipid metabolism, inflammatory responses, and energy homeostasis, and may therefore indirectly contribute to metabolic remodeling in heart failure (50, 51). Nevertheless, whether this pathway directly enhances myocardial fatty acid oxidation and improves heart failure requires further validation in heart-specific experimental models. Taken together, AMPK signaling may represent a potential convergence point through which multiple gut-derived metabolites could be linked to myocardial mitochondrial biogenesis and energy metabolism. However, its direct role across different heart failure phenotypes remains to be further clarified.

### 4.2 Distinct roles of SIRT1/PGC-1*α* and SIRT3-related pathways in metabolite-linked mitochondrial regulation

The SIRT1/PGC-1*α* axis is an important pathway regulating myocardial mitochondrial oxidative metabolism and mitochondrial biogenesis (52). However, direct evidence supporting a specific “gut-derived metabolite–SIRT1/PGC-1*α*–heart failure” signaling cascade remains relatively limited. It is important to distinguish SIRT1/PGC-1*α*-related mitochondrial biogenesis from SIRT3-related mitochondrial antioxidant and metabolic regulation when interpreting metabolite-linked mitochondrial mechanisms. SIRT1 is classically described as a predominantly nuclear/cytosolic NAD+-dependent deacetylase that can regulate PGC-1*α*-mediated transcriptional programs involved in mitochondrial biogenesis (53). In contrast, SIRT3 is a mitochondrial deacetylase that regulates mitochondrial antioxidant defense and metabolic enzymes involved in redox homeostasis and fatty acid oxidation (54). Therefore, evidence supporting SIRT1/PGC-1*α*-related mitochondrial biogenesis should not be merged with evidence supporting SIRT3-related mitochondrial antioxidant or metabolic regulation. Studies in heart failure models have shown that activation of AMPK/SIRT1/PGC-1*α* signaling can restore mitochondrial homeostasis, as reflected by reduced mitochondrial ROS production, improved ATP generation, and attenuation of heart failure progression, indicating the intrinsic cardioprotective significance of this axis (55).

Regarding gut-derived metabolites, SCFAs can directly participate in energy metabolism in the failing myocardium. In hearts with transverse aortic constriction (TAC)-induced failure, butyrate and other SCFAs are more readily oxidized than ketone bodies, suggesting that they may serve as alternative carbon sources to support mitochondrial oxidative metabolism. However, as discussed above, this finding should be interpreted as evidence that failing hearts retain the capacity to oxidize SCFAs under experimental conditions, rather than definitive proof that circulating SCFAs provide quantitatively important fuel in human heart failure. Moreover, this study did not directly demonstrate that these effects are mediated through SIRT1/PGC-1*α* signaling (16). IPA is another representative gut-derived metabolite. *In vitro* and *ex vivo* heart studies have shown that IPA can modulate mitochondrial respiration in cardiomyocytes and alter cardiac function (56). In a heart failure with preserved ejection fraction (HFpEF) model, IPA supplementation improved diastolic function and attenuated myocardial metabolic abnormalities by enhancing NAD+ salvage synthesis and SIRT3-related mitochondrial function (33). Thus, IPA-related evidence in this context primarily supports NAD+/SIRT3-associated mitochondrial antioxidant and metabolic regulation rather than direct activation of the SIRT1/PGC-1*α* mitochondrial biogenesis axis. Conversely, TMAO has been shown to inhibit mitochondrial complex IV activity and reduce myocardial ATP and phosphocreatine levels in heart failure models, thereby aggravating cardiac dysfunction (57). Nevertheless, the myocardial intracellular accumulation, mitochondrial accessibility, and concentration dependence of TMAO require further clarification before this mechanism can be generalized to human heart failure. Taken together, current evidence indicates that gut-derived metabolites may influence mitochondrial oxidative metabolism through distinct and incompletely overlapping pathways. SIRT1/PGC-1*α*-related mitochondrial biogenesis and SIRT3-related mitochondrial antioxidant or metabolic regulation should therefore be discussed separately. Whether specific gut-derived metabolites directly regulate either pathway in human heart failure remains to be established by heart failure-specific mechanistic studies.

### 4.3 Mitochondrial dynamics and quality-control markers: direct and model-dependent evidence

Impaired mitochondrial dynamics is a key manifestation of disrupted mitochondrial homeostasis in heart failure, involving abnormalities in mitochondrial fission, fusion, and quality control. Among these processes, DRP1-mediated mitochondrial fission has received considerable attention in cardiovascular remodeling and myocardial injury (28, 47, 58, 59). However, evidence involving mitochondrial dynamics should be interpreted in a cell-type- and model-specific manner, because some studies evaluate cardiomyocytes whereas others focus on non-cardiomyocyte populations such as macrophages or whole myocardial tissue. Among gut-derived metabolites, relatively direct but model-dependent evidence is available for IPA. In a doxorubicin (DOX)-induced cardiotoxicity model, fecal microbiota transplantation (FMT) altered the gut microbiota and serum metabolite profiles, and correlation analyses suggested a close association between IPA and cardiac function. Both FMT and IPA promoted Nrf2 nuclear translocation, reduced ROS production, and inhibited excessive mitochondrial fission, thereby preserving mitochondrial function and alleviating myocardial injury (60).

It should be noted that direct evidence linking IPA to the regulation of mitochondrial dynamics proteins such as DRP1 and OPA1 mainly comes from DOX-induced cardiotoxicity models. Evidence in HFpEF or pressure overload-induced heart failure remains limited and requires further validation. In an HFpEF model, IPA levels were decreased in both plasma and myocardium; IPA supplementation improved diastolic function, oxidative stress, inflammation, and myocardial NAD+/SIRT3 signaling, suggesting that gut-derived indole metabolites may attenuate HFpEF-related dysfunction by supporting mitochondrial homeostasis in selected models (33). Conversely, the choline/TMAO axis can exacerbate pressure overload-induced heart failure, indicating that different gut-derived metabolites may exert context-dependent effects on myocardial mitochondria and heart failure progression (61). Collectively, gut-derived metabolites may contribute to myocardial injury and heart failure progression by modulating mitochondrial dynamics and related quality-control processes. However, whether they directly regulate mitochondrial dynamics proteins such as DRP1 and OPA1 across different heart failure phenotypes remains to be further investigated.

### 4.4 Mitophagy, apoptosis, and fibrotic remodeling: evidence from selected models

Mitophagy and apoptosis are closely interconnected in heart failure, but heart failure-specific evidence demonstrating that gut-derived metabolites directly regulate the interaction between these two processes remains limited. Much of the evidence linking mitophagy to heart failure is derived from general cardiac stress models rather than gut-metabolite-specific interventions. In a pressure overload-induced heart failure model, Drp1-dependent mitophagy was transiently activated at an early stage and subsequently declined. Restoration of mitophagy alleviated mitochondrial dysfunction and heart failure progression, suggesting that appropriate mitophagy exerts cardioprotective effects (62). AMPK*α*2 can enhance mitophagy by promoting PINK1 phosphorylation, thereby delaying the development of heart failure and further supporting the protective role of PINK1-related mitochondrial quality control in heart failure (63). On the other hand, BNIP3 is considered a marker associated with both mitochondrial death and mitophagy. Its upregulation in pathological hypertrophy and heart failure suggests that, under severe stress conditions, mitophagy may be co-activated with pro-apoptotic signaling (64).

Among gut-derived metabolites, urolithin A is a representative regulator of mitochondrial quality control. Preclinical studies have shown that urolithin A has the potential to improve cardiac function-related parameters and enhance mitochondrial quality control (65). In a doxorubicin-induced cardiotoxicity model, urolithin A enhanced PINK1-regulated mitophagy through Ambra1 and exhibited antioxidant and anti-apoptotic effects, providing direct experimental evidence for the concept that enhancement of effective mitophagy can suppress cardiomyocyte death (66). However, the translational interpretation of urolithin A should consider interindividual differences in urolithin-producing capacity, because gut microbial metabotype may determine whether dietary ellagitannin precursors are efficiently converted into urolithin A. In contrast, the choline/TMAO axis can aggravate pressure overload-induced heart failure, and TMAO can impair myocardial mitochondrial energy metabolism (61). Overall, these findings suggest that gut-derived metabolites may influence mitochondrial homeostasis, cell death, and fibrotic remodeling in a context-dependent manner rather than in uniformly protective or deleterious directions. The strength of evidence varies substantially across metabolites, models, and mitochondrial readouts, and human heart failure-specific confirmation remains limited.

## 5 Pathological and clinical relevance of gut-derived metabolites in heart failure phenotypes

### 5.1 Gut-derived metabolites and myocardial energy metabolic remodeling

Gut-derived metabolites may contribute to energy metabolic remodeling in heart failure by altering myocardial substrate utilization and mitochondrial oxidative capacity. The failing myocardium is typically characterized by impaired fatty acid oxidation, reduced mitochondrial ATP production, and increased reliance on alternative fuels. Studies have shown that, in pressure overload-induced failing hearts, SCFAs can serve as oxidative substrates for the myocardium, with oxidation rates even exceeding those of ketone bodies, suggesting that failing hearts retain the capacity to oxidize SCFAs under experimental conditions (16). However, as discussed above, whether circulating SCFAs provide quantitatively important alternative fuel in human heart failure remains uncertain and may depend on systemic exposure, first-pass metabolism, and myocardial uptake (67). Indole metabolites also exert direct metabolic effects on the myocardium. Gesper et al. screened 25 gut-derived metabolites and found that IPA modulated mitochondrial respiration in cardiomyocytes, acutely enhanced maximal mitochondrial respiratory capacity, and increased left ventricular contractile function in isolated mouse hearts, indicating that this metabolite can directly influence myocardial energy metabolism and cardiac performance (56). In an HFpEF model, IPA levels were reduced, whereas supplementation improved diastolic function by enhancing NAD+ salvage synthesis and SIRT3-related mitochondrial function, further supporting a link between gut-derived metabolites, mitochondrial energy metabolism, and heart failure phenotypes (33). In contrast, choline-derived TMAO exacerbates pressure overload-induced heart failure (61). Further mechanistic studies have shown that TMAO-dependent mechanisms can inhibit mitochondrial complex IV activity, reduce myocardial ATP and phosphocreatine levels, and thereby promote myocardial energy metabolic dysfunction (57). Nevertheless, the relevance of these findings to human heart failure requires cautious interpretation because myocardial TMAO accumulation, intracellular mitochondrial accessibility, exposure duration, and concentration dependence remain incompletely defined. Thus, gut-derived metabolites may modify myocardial energy metabolism through substrate availability or mitochondrial regulatory pathways, but these effects should be interpreted as context-dependent rather than uniformly protective or harmful.

### 5.2 Gut-derived metabolites, myocardial oxidative stress, and inflammation

Gut-derived metabolites may participate in heart failure progression by regulating myocardial oxidative stress and inflammatory responses. Chronic heart failure is intrinsically accompanied by increased oxidative stress and inflammation, with mitochondrial dysfunction serving as a major pathological basis (68). Among currently studied metabolites, IPA represents one of the more directly supported examples of a metabolite linked to mitochondrial redox regulation in selected heart failure-related models. In an HFpEF model, IPA levels were reduced in both plasma and myocardial tissue. IPA supplementation improved diastolic function and attenuated oxidative stress, inflammation, and metabolic remodeling by restoring myocardial NAD+/NADH balance and SIRT3-related mitochondrial function, suggesting that IPA may be involved in a gut microbiota–mitochondrial redox regulation–inflammation axis (33). Conversely, the choline/TMAO axis appears to exert adverse effects under chronic high-choline or high-TMAO exposure conditions. A high-choline diet aggravated cardiac dysfunction, myocardial fibrosis, and inflammation in HFpEF mice, whereas inhibition of gut microbial TMA production with 3,3-dimethyl-1-butanol alleviated these changes (69). In pressure overload-induced heart failure, the TMAO-related pathway is also associated with increased myocardial remodeling and inflammation, while reducing TMAO production improves cardiac remodeling (61). In addition, increasing SCFA-producing gut bacteria and intestinal SCFA levels attenuates TAC-induced left ventricular remodeling, suggesting that SCFA-related pathways may modulate inflammation and gut–heart axis homeostasis (70). However, these findings should not be taken to indicate a simple directional classification of gut-derived metabolites.

### 5.3 Gut-derived metabolites, cardiomyocyte apoptosis, and fibrosis

Gut-derived metabolites may participate in pathological cardiac remodeling in heart failure by influencing cardiomyocyte apoptosis and myocardial fibrosis. Evidence supporting adverse remodeling is relatively stronger for chronic activation of the choline/TMAO pathway in animal models. In pressure overload-induced heart failure, a high-choline diet and its gut microbial metabolite TMAO aggravate cardiac dysfunction and myocardial fibrosis, suggesting that TMAO is not only a risk marker for heart failure but may also participate in cardiac remodeling (61). In HFpEF mice, a high-choline diet similarly worsens diastolic dysfunction, myocardial fibrosis, and inflammation, whereas inhibition of gut microbial TMA production with 3,3-dimethyl-1-butanol (DMB) attenuates these abnormalities, further supporting a potential causal contribution of gut microbial TMA production to fibrosis and heart failure-related remodeling in experimental models (69). Mechanistically, TMAO can induce cardiac hypertrophy and fibrosis through pathways involving Smad3 signaling, suggesting that it may promote fibroblast activation and collagen deposition (71). With respect to apoptosis, gut-derived indoxyl sulfate provides emerging direct evidence. In patients with chronic kidney disease complicated by heart failure, *Escherichia coli* and its derived metabolite indoxyl sulfate are elevated, and mechanistic experiments suggest that indoxyl sulfate impairs myocardial mitochondrial function and induces cardiomyocyte apoptosis through the AHR–CYP1B1 axis, thereby promoting heart failure progression (38). However, because indoxyl sulfate is highly protein-bound and strongly influenced by renal dysfunction, the free biologically active concentration and CKD-specific context should be considered when extrapolating these findings to broader heart failure populations. In contrast, urolithin A has been associated with reduced myocardial fibrosis, improved cardiac function, and enhanced mitochondrial quality control in aging- and heart failure-related models (72). Nevertheless, urolithin A-related effects may depend on host urolithin-producing metabotype and may not be uniformly achieved through dietary ellagitannin precursors. Therefore, different gut-derived metabolites may shape myocardial remodeling through apoptosis, fibrosis, inflammation, or mitochondrial quality-control pathways, but the direction and magnitude of these effects remain dependent on biological context, disease model, and metabolite exposure.

### 5.4 Associations between gut-derived metabolites and clinical phenotypes of heart failure

The associations between gut-derived metabolites and clinical phenotypes of heart failure are mainly reflected in the presence of heart failure, ejection fraction-based classification, New York Heart Association (NYHA) functional class, and prognostic risk. Clinical studies have shown that the gut microbiota-derived metabolite TMAO is elevated in patients with heart failure and is associated with NYHA class, ischemic etiology, and adverse outcomes, suggesting that it may reflect disease severity and clinical risk (21). Further studies comparing heart failure with reduced ejection fraction (HFrEF) and HFpEF have found that the prognostic value of TMAO may differ across ejection fraction phenotypes, indicating that TMAO may not only serve as a general heart failure biomarker but also contribute to the distinction of clinical heterogeneity in heart failure (73). In HFpEF populations, TMAO has been reported as an independent risk factor, supporting its clinical association with diastolic dysfunction-related heart failure phenotypes (74). In addition to TMAO, PAGln is another important gut-derived metabolite. Studies have shown that PAGln is associated with the presence of heart failure, disease severity, and increased risk of cardiovascular mortality, and may contribute to heart failure progression through gut microbiota-related metabolic mechanisms (41, 42). However, clinical associations involving TMAO, PAGln, and indoxyl sulfate should be interpreted cautiously because renal dysfunction, heart failure severity, altered renal clearance, and reverse causality may contribute to elevated circulating metabolite levels. In particular, heart failure itself can promote intestinal hypoperfusion, venous congestion, barrier dysfunction, endotoxin translocation, and gut microbial dysbiosis, making it difficult to distinguish gut-to-heart causality from heart-to-gut secondary changes.

Conversely, evidence related to SCFAs suggests that better clinical recovery after new-onset heart failure is associated with reversal of gut dysbiosis and increased SCFA production, indicating that SCFA-related microbial metabolic profiles may be linked to milder or more reversible clinical phenotypes (67). Nevertheless, this observation remains associative and does not prove that increasing circulating SCFAs directly restores myocardial mitochondrial function. Therefore, gut-derived metabolites may serve as metabolic biomarkers linking gut microbiota dysbiosis with clinical heterogeneity in heart failure. At present, their value as causal mediators or therapeutic targets requires further validation through longitudinal cohorts, renal-function-stratified analyses, direct myocardial or circulating free-metabolite measurements, and intervention studies with mitochondrial or metabolic endpoints. Evidence linking gut-derived metabolites to myocardial mitochondrial homeostasis and heart failure phenotypes across experimental and clinical settings is summarized in Table 2.

**Table 2:** Evidence map linking gut-derived metabolites to mitochondrial regulation across models and HF phenotypes.

Evidence context	Metabolite or pathway	Model or population	Cell/tissue context	Main finding	Mitochondrial evidence level	Major limitation	Current interpretation	Key references
Chronic HF cohorts	TMAO	Human chronic HF cohorts and meta-analysis.	Circulating metabolite.	Higher TMAO is associated with HF severity and adverse outcomes.	Clinical association only.	Observational design, renal confounding, reverse causality, and no direct myocardial mitochondrial readout.	Useful risk biomarker; causal role remains uncertain.	(21, 22, 44)
HFrEF and HFpEF cohorts	TMAO	Human ejection-fraction phenotype cohorts.	Circulating metabolite.	Prognostic relevance may differ between HFrEF and HFpEF.	Clinical association only.	Phenotype heterogeneity, renal function, treatment confounding, and uncertain myocardial exposure.	Phenotype-sensitive biomarker requiring cautious interpretation.	(73, 74)
Acute experimental exposure	TMAO	Isolated cardiac muscle/acute exposure model.	Cardiac muscle preparations.	Acute TMAO increased contractility in Oakley et al.; this should not be interpreted as chronic contractile impairment.	No direct myocardial mitochondrial readout.	Acute exposure and non-HF context; not evidence of TMAO-induced HF dysfunction.	Context warning for exposure-duration dependence.	(25)
HFpEF experimental model	IPA	Animal HFpEF model.	Myocardial tissue and cardiomyocyte-related readouts.	IPA supplementation improved diastolic function, oxidative stress, inflammation, NAD+ salvage, and SIRT3-related signaling.	Indirect mitochondrial signaling evidence.	Animal model only; human HFpEF validation and dose/exposure translation needed.	Promising phenotype-specific preclinical evidence.	(33)
Cardiomyocyte/*ex vivo* studies	IPA	Cardiomyocytes and isolated mouse hearts.	Cardiomyocytes and *ex vivo* myocardium.	IPA modulated mitochondrial respiration and acute cardiac function.	Direct mitochondrial functional evidence.	Acute exposure and uncertain chronic *in vivo* HF relevance.	Strong mechanistic evidence needing translational validation.	(56)
Pressure-overload HF	SCFAs	TAC-induced failing hearts.	*Ex vivo* perfused myocardium.	SCFAs can be oxidized by failing hearts under controlled substrate conditions.	Direct mitochondrial functional evidence.	*Ex vivo* setting; uncertain circulating exposure, myocardial uptake, and quantitative human contribution.	Mechanistically informative but exposure-limited.	(16, 35)
Pressure-overload/HFpEF models	Choline-TMAO pathway	Mouse pressure-overload and HFpEF-related models.	Whole myocardium.	High choline/TMAO worsened remodeling; TMA-lowering interventions improved selected remodeling outcomes.	Preclinical intervention evidence.	Animal model only; dose, renal handling, and acute/chronic exposure ambiguity.	Preclinical causal support, not clinical proof.	(57, 61, 69, 80–82)
CKD-associated HF	Indoxyl sulfate	CKD-HF mechanistic models.	Myocardium/cardiomyocytes.	Indoxyl sulfate was linked to mitochondrial dysfunction and apoptosis through AHR-CYP1B1-related mechanisms.	Indirect mitochondrial signaling evidence.	CKD-specific context, protein binding, free fraction, and renal dependence.	Cardiorenal mechanism with limited generalizability.	(36–38, 40)
DOX cardiotoxicity model	IPA or FMT-associated metabolite changes	DOX-induced cardiotoxicity model.	Myocardium/cardiomyocyte injury context.	FMT/IPA reduced ROS and excessive mitochondrial fission markers.	Direct mitochondrial structural evidence.	Non-HF model; cell-type ambiguity and HF phenotype validation needed.	Mechanistic support, not classical HF evidence.	(60)
Urolithin A preclinical models	Urolithin A	Preclinical cardiac injury, aging, or fibrosis-related models.	Myocardium/cardiomyocytes.	Urolithin A enhanced mitochondrial quality-control or mitophagy-related readouts and reduced fibrosis/apoptosis in models.	Direct mitochondrial structural evidence.	Preclinical evidence; host metabotype, bioavailability, and human HF relevance uncertain.	Mechanistically promising but clinically unproven in HF.	(39, 65, 66, 72)
HF clinical cohorts	PAGln	Human HF cohorts.	Circulating metabolite.	PAGln is associated with HF severity, cardiovascular events, and prognosis.	Clinical association only.	Observational design, renal-metabolic confounding, reverse causality, and lack of direct mitochondrial endpoints.	Risk marker with unvalidated mitochondrial mechanism.	(40–43)

Direct mitochondrial evidence was considered present when studies reported mitochondrial respiration, OXPHOS, ATP production, mitochondrial membrane potential, mitochondrial ROS, mitochondrial morphology, mitophagy-related readouts, or mitochondrial complex activity. Otherwise, evidence was classified as indirect, preclinical intervention-based, or associative. AHR, aryl hydrocarbon receptor; ATP, adenosine triphosphate; CKD, chronic kidney disease; CYP1B1, cytochrome P450 family 1 subfamily B member 1; DOX, doxorubicin; FMT, fecal microbiota transplantation; HF, heart failure; HFpEF, heart failure with preserved ejection fraction; HFrEF, heart failure with reduced ejection fraction; IPA, indole-3-propionic acid; NAD+, nicotinamide adenine dinucleotide; OXPHOS, oxidative phosphorylation; PAGln, phenylacetylglutamine; ROS, reactive oxygen species; SCFAs, short-chain fatty acids; TAC, transverse aortic constriction; TMAO, trimethylamine N-oxide.

## 6 Therapeutic strategies targeting gut microbiota-derived metabolic pathways: promise and translational limitations

### 6.1 Modulation of gut-derived metabolite profiles by probiotics and prebiotics

Probiotics and prebiotics may influence the gut–heart axis in heart failure by reshaping the gut microbiota and its metabolite profiles. Available experimental evidence in this area has mainly focused on SCFA-related pathways and endotoxin-related profiles. In an angiotensin II-induced model of cardiovascular injury, a high-fiber diet or acetate supplementation altered the gut microbiota, and reintroduction of SCFAs attenuated hypertension, cardiac hypertrophy, and fibrosis. These effects were associated with SCFA-sensing receptors such as GPR43 and GPR109A (75). In female rats with experimentally induced heart failure, a prebiotic complex containing fermented wheat bran and inactivated yeast reduced circulating endotoxin levels, Lactobacillus markers, and opportunistic pathogen markers, suggesting that prebiotics may correct heart failure-related gut dysbiosis and endotoxemia (76).

However, not all prebiotic interventions are effective. In a genetic model of dilated cardiomyopathy, a high-fiber diet altered the gut microbiota but failed to improve cardiac remodeling, cardiomyocyte apoptosis, or hemodynamic parameters, indicating that the efficacy of such interventions may depend on the etiology of heart failure (77). Evidence for probiotics in humans remains even more cautious. The randomized GutHeart trial showed that three months of treatment with *Saccharomyces boulardii* or rifaximin did not significantly improve left ventricular ejection fraction, gut microbial diversity, TMAO levels, or systemic inflammation (78). This negative randomized trial is an important cautionary signal for the field. It does not necessarily refute the gut–heart axis, but it indicates that broad and non-personalized microbiome modulation may be insufficient to improve established HFrEF, particularly when interventions are not guided by baseline metabolite profiles, microbial metabotypes, renal function, heart failure phenotype, or direct mitochondrial endpoints. These negative findings highlight the need to distinguish microbiome modulation from metabolite-targeted intervention. Therefore, the most reliable conclusion at present is that prebiotics and dietary fiber have relatively stronger evidence for modulating SCFA production and endotoxin-related profiles, whereas clinical evidence supporting probiotic-mediated regulation of TMAO and other metabolites in patients with heart failure remains insufficient. Future trials should consider longer intervention duration, metabolite-guided patient selection, renal-function stratification, microbiome/metabotype stratification, and predefined metabolic or mitochondrial readouts.

### 6.2 Dietary intervention, metabolite exposure, and myocardial mitochondrial metabolism

Dietary interventions may affect mitochondrial energy metabolism in the failing heart by altering gut-derived metabolite profiles, particularly SCFA availability (16, 79). The most direct evidence comes from dietary fiber and SCFAs. In a mineralocorticoid-induced model of hypertensive cardiac injury, a high-fiber diet reshaped the gut microbiota and increased the abundance of acetate-producing bacteria, and both high-fiber feeding and acetate supplementation reduced blood pressure, left ventricular hypertrophy, and myocardial fibrosis (79).

From the perspective of myocardial mitochondrial metabolism, hearts failing after TAC exhibit impaired long-chain fatty acid oxidation (16). *Ex vivo* perfusion experiments showed that butyrate, a representative gut-derived SCFA, can bypass the limitation imposed by carnitine palmitoyltransferase 1 (CPT1) and is more readily oxidized than ketone bodies in the failing heart, suggesting that it may serve as a potential alternative energy substrate (16). However, this observation should be interpreted as evidence of myocardial oxidative capacity under experimental conditions rather than definitive proof that dietary fiber intervention generates sufficient circulating or myocardial SCFA exposure to fuel the human failing heart. In contrast, diets rich in choline, phosphatidylcholine, or carnitine can be metabolized by gut microbes to TMA, which is subsequently converted to TMAO. TMAO has been implicated in the progression of heart failure (14). In a pressure overload-induced heart failure model, dietary choline and TMAO supplementation exacerbated heart failure severity (61). Further mechanistic work showed that TAC or myocardial infarction may induce brown adipose tissue dysfunction and increase TMAO levels. Exogenous TMAO inhibited myocardial mitochondrial complex IV activity and reduced cardiac ATP and phosphocreatine levels, whereas lowering TMAO through flavin-containing monooxygenase inhibition improved pressure overload-induced cardiac dysfunction (57). Nevertheless, dietary modulation of the choline–TMAO pathway should be interpreted cautiously because circulating TMAO is influenced by renal clearance, host FMO3 activity, gut microbial composition, dietary pattern, and exposure duration. Thus, dietary factors may modify myocardial energy metabolism by altering SCFA availability or the choline–TMAO axis, but their effects are unlikely to be uniform across patients and should be viewed as context-dependent rather than simply protective or harmful.

### 6.3 Development of drugs targeting gut microbial enzymes

Among pharmacological strategies targeting gut microbial enzymes, inhibition of microbial TMA production currently has relatively strong preclinical evidence in the field of heart failure. Dietary substrates such as choline and carnitine can be metabolized by gut microbial enzymes, including choline TMA-lyase, to generate TMA, which is subsequently converted to TMAO in the liver. In pressure overload-induced heart failure, dietary choline or TMAO supplementation worsened heart failure severity, suggesting that this metabolic axis is a potential preclinical intervention target (61).

3,3-Dimethyl-1-butanol (DMB), a structural analog of choline, was initially identified as a non-lethal inhibitor of gut microbial TMA production and has since been used as a tool compound to interrogate the TMA/TMAO pathway (80). In mice with aortic constriction-induced heart failure, DMB reduced plasma TMAO levels and attenuated myocardial hypertrophy, fibrosis, inflammation, and electrical remodeling, indicating that targeting gut microbial TMA-generating enzymes may improve pressure overload-induced cardiac remodeling (81). Another study further showed that withdrawal of dietary TMAO or inhibition of gut microbial TMAO generation improved cardiac function and structural remodeling in heart failure mice, supporting nonlethal inhibition of gut microbial TMA production as a promising preclinical strategy for heart failure (82). In an HFpEF model, a high-choline diet aggravated diastolic dysfunction, myocardial fibrosis, and inflammation, whereas DMB alleviated high-choline-associated cardiac injury, suggesting that this strategy may also be relevant to heart failure with preserved ejection fraction (69). Therefore, targeting gut microbial TMA-generating enzymes to reduce TMAO is currently one of the most evidence-supported directions in gut–heart axis-based drug development for heart failure. However, this evidence is still largely preclinical. Whether TMA-lowering strategies improve clinical outcomes, reverse mitochondrial dysfunction, or benefit specific HFpEF or HFrEF subgroups remains unknown. Clinical translation will require careful assessment of safety, durability of microbiome effects, compensatory microbial pathways, renal function, dietary background, and direct metabolic endpoints.

### 6.4 Interventional potential of fecal microbiota transplantation in heart failure-related gut–heart axis research

The value of fecal microbiota transplantation (FMT) in heart failure should currently be viewed more as an experimental tool for validating the causal relationship among the gut microbiota, metabolites, and cardiac function, rather than as an established clinical therapy. In a TAC-induced mouse model of heart failure, heart failure altered the gut microbial structure and fecal metabolite profile. Transplantation of fecal microbiota from heart failure mice into recipient mice induced glucose intolerance, insulin resistance, and increased glucagon levels, suggesting that heart failure-associated microbiota and their metabolites can transmit host metabolic dysfunction (83).

In an obesity-related pre-HFpEF model, FMT improved early systolic and diastolic dysfunction. This effect was associated with improvements in gut-derived butyrate production and branched-chain amino acid catabolic pathways, indicating that gut microbiota remodeling may have interventional potential for early myocardial metabolic abnormalities in HFpEF (84). In another model of post-myocardial infarction cardiac dysfunction, transplantation of microbiota from exercised mice improved cardiac function in recipient mice. The study further identified gut-derived metabolites, including 3-hydroxyphenylacetic acid and 4-hydroxybenzoic acid, which attenuated cardiomyocyte apoptosis and cardiac dysfunction through the NRF2 pathway (85). Taken together, FMT studies suggest that reconstruction of selected microbial communities and metabolite profiles may indirectly improve myocardial metabolism, inflammation, and cell-death processes in heart failure. However, the current direct evidence is mainly derived from animal models, and FMT should not yet be described as a clinically proven therapy for heart failure. In addition, FMT has substantial translational barriers, including donor variability, durability of engraftment, safety concerns, patient selection, regulatory issues, and uncertainty regarding which microbial metabolites mediate cardiac effects. Therefore, FMT is best considered a mechanistic tool for causal validation rather than a near-term therapeutic strategy for heart failure.

Overall, microbiota-targeted interventions may partially restore mitochondrial function in selected experimental models, but whether they can reverse established mitochondrial dysfunction in human heart failure remains unproven. Potential intervention strategies targeting gut microbiota-derived metabolic pathways and their translational limitations are summarized in Table 3.

**Table 3:** Gut microbiota-targeted interventions and translational status in heart failure.

Intervention strategy	Targeted pathway	Evidence basis	Reported cardiac or mitochondrial effect	Major translational limitation	Current translational status	Key references
Dietary fiber/acetate/SCFA-related intervention	SCFA production and SCFA-sensing receptors.	Hypertensive cardiac injury and cardiometabolic models.	Reduced blood pressure, hypertrophy, fibrosis, and inflammatory remodeling in selected models.	Not uniformly HF-specific; exposure, receptor expression, and etiology may determine response.	Preclinical candidate requiring responder stratification.	(75, 79)
Prebiotic intervention	SCFA-producing microbiota and endotoxin-related profiles.	Experimental HF and genetic DCM-related models.	May reduce endotoxemia or shift microbial profiles, but cardiac functional benefit is inconsistent.	Genetic predisposition and HF etiology may override microbiome modulation.	Not clinically established.	(76, 77)
Dietary choline/carnitine modulation	Choline/carnitine-TMA-TMAO pathway.	Animal HF models and TMAO-pathway mechanistic studies.	High choline/TMAO worsened remodeling in selected models; lowering pathway flux may improve outcomes experimentally.	Diet complexity, renal clearance, FMO activity, adherence, and long-term safety are unresolved.	Mechanistically plausible but unproven in HF.	(14, 57, 61, 69)
DMB or microbial TMA-lyase inhibition	Gut microbial TMA production.	Atherosclerosis tool-compound work and HF animal models.	Reduced TMAO and improved hypertrophy, fibrosis, inflammation, electrical remodeling, or function in models.	Preclinical only; human safety, durability, and microbiome effects are unknown.	Promising preclinical candidate; not established therapy.	(69, 80–82)
Host FMO-related TMAO modulation	Host conversion of TMA to TMAO.	Experimental TMAO-pathway studies.	Lowering TMAO formation may improve energetics or remodeling in selected experimental settings.	Direct host-enzyme targeting in HF is less developed and may have systemic metabolic effects.	Mechanistically informative but less clinically developed than microbial TMA-lyase inhibition.	(14, 57, 61)
Saccharomyces boulardii or rifaximin/GutHeart trial	Broad microbiome modulation, TMAO, and inflammation.	Randomized trial in established HFrEF.	No significant improvement in LVEF, gut microbial diversity, TMAO, or systemic inflammation.	Broad non-personalized intervention, established HFrEF, and limited patient-selection strategy.	Negative randomized trial.	(78)
FMT	Whole microbial community and metabolite profile.	Animal HF, pre-HFpEF, and post-MI models.	Can transmit or improve metabolic/cardiac phenotypes depending on donor context.	Donor variability, engraftment durability, safety, regulatory barriers, and undefined active metabolites.	Experimental tool for causal validation; not near-term clinical HF therapy.	(83–85)
Urolithin A supplementation	Mitochondrial quality control and mitophagy.	Preclinical cardiac injury and fibrosis models.	May enhance mitophagy/quality control and reduce apoptosis or fibrosis.	Human HF evidence is lacking; dietary precursor conversion depends on host metabotype.	Mechanistically promising but unproven in HF.	(39, 65, 66, 72)
IPA or tryptophan-derived metabolite modulation	NAD+ salvage, SIRT3-related regulation, redox balance, and inflammation.	HFpEF model, cardiomyocyte, and *ex vivo* studies.	Improved diastolic function and mitochondrial-related metabolic abnormalities in selected models.	Dose, delivery, safety, microbial production, and HF phenotype validation remain unresolved.	Mechanistically promising but requires phenotype-specific validation.	(33, 56, 60)

DCM, dilated cardiomyopathy; DMB, 3,3-dimethyl-1-butanol; FMO, flavin-containing monooxygenase; FMT, fecal microbiota transplantation; HF, heart failure; HFpEF, heart failure with preserved ejection fraction; HFrEF, heart failure with reduced ejection fraction; IPA, indole-3-propionic acid; LVEF, left ventricular ejection fraction; MI, myocardial infarction; NAD^+^, nicotinamide adenine dinucleotide; SCFAs, short-chain fatty acids; SIRT3, sirtuin 3; TMA, trimethylamine; TMAO, trimethylamine N-oxide.

### 6.5 Critical appraisal of the current evidence and translational priorities

Although increasing evidence links gut-derived metabolites to myocardial mitochondrial homeostasis and heart failure, the strength of evidence varies substantially across metabolites, models, and endpoints. At present, the most compelling causal evidence comes from preclinical intervention studies targeting the choline–TMA/TMAO pathway. Dietary choline or TMAO supplementation worsens cardiac dysfunction, fibrosis, inflammatory activation, and energetic impairment in pressure overload- and HFpEF-related models, whereas inhibition of gut microbial TMA production or reduction of TMAO formation improves cardiac remodeling in selected animal models (57, 61, 69, 80–82). These findings support the choline–TMA/TMAO axis as one of the most evidence-supported preclinical therapeutic directions in the gut–heart field. However, clinical evidence for TMAO remains largely associative and is influenced by renal clearance, heart failure severity, dietary background, and reverse causality. Therefore, TMAO should currently be viewed as a context-dependent candidate mediator and biomarker rather than an established therapeutic target in human heart failure.

The evidence for SCFAs, IPA, and urolithin A is mechanistically promising but also requires careful qualification. SCFA oxidation by failing hearts demonstrates that the myocardium retains the capacity to oxidize these substrates under controlled experimental conditions, but it does not prove that circulating SCFAs provide quantitatively important fuel in patients with heart failure (16, 35). IPA has relatively direct cardiomyocyte, *ex vivo*, and HFpEF-model evidence linking it to mitochondrial respiration, NAD+ salvage, SIRT3-related mitochondrial regulation, redox balance, and inflammatory modulation (33, 56, 60). Urolithin A has stronger support in selected preclinical models for mitochondrial quality control, mitophagy-related signaling, anti-apoptotic effects, and anti-fibrotic remodeling (39, 65, 66, 72). Nevertheless, the translational relevance of IPA and urolithin A depends on dose, bioavailability, microbial production, host metabolic status, myocardial exposure, and, for urolithin A, individual urolithin-producing metabotype.

For indoxyl sulfate, PAGln, kynurenine-pathway metabolites, and bile acid dysregulation, current evidence is more limited or more strongly influenced by systemic disease context. Indoxyl sulfate has mechanistic support in CKD-associated heart failure, but its high albumin binding and renal dependence limit direct extrapolation to broader heart failure populations (36–40). PAGln is associated with heart failure presence, disease severity, and adverse outcomes, but direct myocardial mitochondrial mechanisms remain insufficiently established, and renal-metabolic confounding is a major concern (41–43). Kynurenine-pathway metabolites and bile acids may reflect inflammatory, metabolic, hepatic, renal, or congestion-related states rather than direct myocardial mitochondrial injury. Thus, these metabolites may currently be more useful as disease-context biomarkers or hypothesis-generating signals than as validated mitochondrial therapeutic targets.

Overall, the field is limited by three major gaps. First, circulating metabolite concentrations do not necessarily reflect free, bioavailable, intracellular, or myocardial mitochondrial exposure. Second, many studies infer mitochondrial relevance from signaling, inflammatory, apoptotic, or fibrotic markers rather than directly measuring mitochondrial respiration, complex activity, ATP or phosphocreatine levels, membrane potential, mitochondrial ROS, or mitochondrial morphology. Third, clinical associations are difficult to separate from renal dysfunction, heart failure severity, and heart-to-gut reverse causality. Future studies should therefore prioritize metabolite-guided and phenotype-specific designs, renal-function-stratified analyses, direct mitochondrial functional endpoints, and integration of metagenomics, targeted metabolomics, single-cell or spatial omics, and causal intervention experiments. From a translational perspective, broad microbiome modulation alone may be insufficient, as suggested by the negative GutHeart trial (78). More rational strategies should focus on clearly defined microbial enzymes, metabolites, responder metabotypes, myocardial exposure, and HFpEF- or HFrEF-specific mechanisms.

## 7 Conclusions and perspectives

The interaction between gut-derived metabolites and myocardial mitochondrial homeostasis provides a new mechanistic perspective for understanding energy metabolic remodeling, inflammatory activation, and pathological myocardial remodeling in heart failure. Current evidence indicates that different metabolites may exert context-dependent effects on heart failure progression. Short-chain fatty acids can be oxidized by failing myocardium under experimental conditions and may influence intestinal barrier integrity, inflammation, and host metabolic homeostasis; however, whether circulating SCFAs provide quantitatively important fuel for the human failing heart remains uncertain. Tryptophan-derived metabolites, such as IPA, have been shown in HFpEF and cardiomyocyte models to improve NAD+/SIRT3-related mitochondrial function and attenuate oxidative stress and inflammatory responses. Urolithin A has also been associated with potentially favorable mitochondrial quality-control and anti-fibrotic effects in selected models, although its translational potential may depend on individual urolithin-producing metabotypes. In contrast, the choline/TMAO axis, indoxyl sulfate, PAGln, and certain disturbances in bile acid metabolism are associated with heart failure severity, myocardial fibrosis, inflammatory activation, mitochondrial energetic impairment, and adverse prognosis. Nevertheless, these associations should not be interpreted as uniformly causal or detrimental, because metabolite concentration, exposure duration, bioavailability, protein binding, renal clearance, heart failure phenotype, and cellular targets may substantially modify their biological effects. These findings suggest that gut-derived metabolites are not only metabolic biomarkers of heart failure risk and phenotypic heterogeneity, but may also represent context-dependent and still incompletely validated intervention targets for modulating myocardial mitochondrial homeostasis and heart failure progression.

However, substantial evidence gaps remain in this field. First, some mechanistic studies have been derived from models of ischemia–reperfusion injury, doxorubicin-induced cardiotoxicity, metabolic diseases, or hepatic and renal disorders, and therefore cannot be directly extrapolated to typical HFrEF, HFpEF, or pressure overload-induced heart failure. Second, the biological effects of different metabolites may depend on their concentration, exposure duration, host metabolic status, renal function, dietary pattern, and heart failure phenotype. Therefore, a simple classification of these metabolites as either “protective” or “detrimental” is insufficient. Third, circulating total metabolite concentrations do not necessarily reflect free, bioavailable, intracellular, or myocardial mitochondrial exposure. This issue is particularly important for protein-bound or renally cleared metabolites such as indoxyl sulfate, PAGln, and TMAO. Fourth, clinical associations may be influenced by cardiorenal confounding and reverse causality, because heart failure itself can promote intestinal hypoperfusion, venous congestion, barrier dysfunction, dysbiosis, and altered metabolite profiles. Fifth, many reported mitochondrial effects remain cell-type-specific or indirectly inferred, and future studies should distinguish cardiomyocyte-autonomous mechanisms from effects mediated by fibroblasts, endothelial cells, macrophages, or systemic inflammation.

Several areas, including bile acid signaling, the SIRT1/PGC-1*α* axis, mitochondrial dynamics proteins, mitophagy, and fecal microbiota transplantation, still lack sufficient heart failure-specific evidence and direct evidence related to myocardial mitochondrial regulation. Future studies should integrate pressure overload models, post-ischemic heart failure models, HFpEF and HFrEF models, and prospective clinical cohorts, together with metagenomics, targeted metabolomics, myocardial mitochondrial functional assays, and causal intervention experiments. Multiomics and artificial intelligence-assisted analyses may help identify microbial-metabolite-host networks, prioritize candidate metabolites, define responder subgroups, and generate testable hypotheses, but these approaches must be validated by mechanistic experiments and prospective clinical studies. Such integrated approaches will be essential to clarify the causal roles, dose–response relationships, cellular targets, and phenotype-specific effects of key gut-derived metabolites. Only after metabolite exposure, mechanistic causality, mitochondrial readouts, renal confounding, and clinical benefit are rigorously validated should gut microbiota- or metabolite-targeted strategies be rationally tested as phenotype-specific adjunctive approaches in heart failure.
