# Supplementary material for: Gut-derived metabolites and mitochondrial homeostasis in heart failure: mechanistic links and translational perspectives
Source: Front Cardiovasc Med. 2026 Aug 3;13:1917012. doi: 10.3389/fcvm.2026.1917012 (PMC13478550; doi:10.3389/fcvm.2026.1917012)
Supplement: Supplementary file 1 [file Table1.docx]

**Supplementary Table S1. Translational constraints for major gut-derived metabolites and community-level gut dysbiosis discussed in this review.**

| **Metabolite or pathway** | **Main source or precursor** | **Circulating exposure consideration** | **Protein binding or bioavailability** | **Renal dependence** | **Experimental exposure concern** | **Direct myocardial or mitochondrial evidence status** | **Key translational caveat** | **Key references** |
| --- | --- | --- | --- | --- | --- | --- | --- | --- |
| Short-chain fatty acids | Dietary fiber fermentation by gut microbiota; acetate, propionate, and butyrate. | Intestinal luminal concentrations are much higher than peripheral circulating concentrations; circulating exposure may not reflect myocardial exposure. | Butyrate is substantially consumed by colonocytes and affected by splanchnic or hepatic first-pass handling; systemic availability is limited compared with intestinal exposure. | Not primarily interpreted as renally retained uremic solutes, but circulating levels may still vary with systemic metabolic state and diet. | Ex vivo perfusion studies demonstrate myocardial oxidative capacity under controlled substrate conditions; this does not prove quantitatively important fuel contribution in human HF. | Direct ex vivo substrate oxidation evidence in failing hearts; limited direct human myocardial mitochondrial evidence. | SCFA-related effects may depend on systemic exposure, myocardial uptake, receptor expression, gut barrier effects, and HF etiology. | (15, 16, 35, 70, 75, 76, 79) |
| Trimethylamine N-oxide pathway | Dietary choline, phosphatidylcholine, and carnitine are converted by gut microbes to TMA, which is converted by hepatic FMO enzymes to TMAO. | Circulating TMAO reflects diet, microbial TMA production, hepatic FMO activity, and renal clearance; plasma levels do not necessarily prove myocardial mitochondrial exposure. | TMAO is a small circulating metabolite, but myocardial intracellular accumulation and mitochondrial accessibility remain incompletely defined. | Strongly influenced by renal clearance; eGFR and CKD may confound clinical associations. | Experimental effects may differ between acute and chronic exposure; Oakley et al. reported acute positive inotropic effects, whereas chronic high exposure worsens remodeling in selected models. | Animal and mechanistic studies report complex IV, ATP, or phosphocreatine-related effects; clinical data are largely associative. | TMAO should be interpreted as a context-dependent metabolite rather than uniformly harmful; causality is complicated by renal confounding and reverse causality. | (21, 22, 25, 44, 57, 61, 69, 80, 81, 82) |
| Bile acid dysregulation | Gut microbiota-liver bile acid metabolism; primary and secondary bile acids. | Bile acid profiles reflect hepatic function, intestinal metabolism, microbial conversion, and enterohepatic circulation. | Bioactivity depends on bile acid species, receptor affinity, conjugation status, and tissue distribution. | Not primarily a renal-retention metabolite, but bile acid profiles may be altered by systemic, hepatic, metabolic, or HF-related congestion states. | Experimental bile acid excess or receptor activation may not reflect physiological bile acid profiles in human HF. | Clinical HF evidence is mainly associative; experimental data support cardiometabolic effects, but direct HF-specific myocardial mitochondrial readouts remain limited. | Effects are bile-acid-subtype and receptor-specific; direct mitochondrial relevance in HF remains incompletely established. | (26, 27, 50, 51) |
| Tryptophan-derived metabolites / indole-3-propionic acid | Microbial tryptophan metabolism. | Circulating and myocardial IPA levels may vary with microbial composition, substrate availability, diet, and HF phenotype. | Bioavailability and tissue exposure may depend on microbial production, host absorption, and systemic metabolism; direct myocardial exposure should be verified in mechanistic studies. | Not primarily discussed as a classic protein-bound uremic toxin, but tryptophan metabolites may be influenced by systemic inflammation, renal function, and host metabolic state. | In vitro or ex vivo IPA exposure may not reproduce chronic in vivo production or tissue levels. | Cardiomyocyte and ex vivo evidence supports mitochondrial respiration effects; HFpEF animal evidence supports NAD+ salvage and SIRT3-related mitochondrial regulation. | IPA appears mechanistically promising, but dose, exposure, microbial production, and human HF validation remain uncertain. | (33, 56, 60) |
| Kynurenine pathway metabolites | Host and microbial tryptophan metabolism; inflammation-related activation of tryptophan catabolism. | Plasma kynurenines may reflect immune activation, systemic inflammation, renal function, and catabolic state. | Bioactivity differs across kynurenine metabolites; tissue-specific exposure and myocardial mitochondrial effects are not fully defined. | Some kynurenine metabolites may be influenced by renal function and systemic disease burden. | Clinical prognostic associations do not establish direct myocardial mitochondrial regulation. | Clinical prognostic association; limited direct myocardial mitochondrial evidence in HF. | Kynurenines may serve more as inflammatory-metabolic risk markers than validated mitochondrial therapeutic targets in HF. | (30, 34) |
| Indoxyl sulfate | Gut microbial indole production from tryptophan followed by hepatic sulfation; protein-bound uremic toxin. | Total plasma concentration may substantially differ from free biologically active concentration. | Highly albumin-bound; free fraction and albumin status are important for biological interpretation. | Strongly dependent on renal clearance; CKD context is central. | In vitro studies using total concentrations without albumin may overestimate free cardiomyocyte exposure. | CKD-HF mechanistic evidence links indoxyl sulfate to mitochondrial dysfunction and cardiomyocyte apoptosis through AHR-CYP1B1-related mechanisms. | Findings should be extrapolated cautiously to non-CKD HF populations; free concentration and renal function should be considered. | (36, 37, 38, 40) |
| Urolithin A | Gut microbial metabolism of ellagitannins. | Exposure after dietary precursors varies substantially across individuals depending on urolithin-producing metabotype. | Direct supplementation may differ from food-based precursor strategies; conversion from ellagitannins is microbiota-dependent. | Not primarily interpreted as a renally cleared uremic solute, but systemic exposure may vary with host metabolism and gut microbial capacity. | Preclinical supplementation may not represent dietary precursor exposure in non-producers or low producers. | Preclinical evidence supports mitochondrial quality control, mitophagy-related signaling, anti-apoptotic, or anti-fibrotic effects; human HF-specific evidence is limited. | Host metabotype is a major translational determinant; not all patients may generate sufficient urolithin A from diet. | (39, 65, 66, 72) |
| Phenylacetylglutamine | Microbial phenylalanine metabolism. | Circulating PAGln is associated with HF presence, disease severity, and cardiovascular mortality, but circulating levels may reflect renal-metabolic risk. | Direct myocardial bioavailability and mitochondrial accessibility are insufficiently established. | Renal function and uremic solute biology are important interpretive factors. | Clinical association does not prove direct myocardial mitochondrial toxicity or therapeutic targetability. | Clinical association evidence is stronger than direct mitochondrial evidence. | PAGln may be a useful microbial-metabolic risk marker, but causal mitochondrial mechanisms in HF remain insufficiently validated. | (40, 41, 42, 43) |
| Gut microbial community-level dysbiosis | Altered gut microbial composition, intestinal barrier dysfunction, endotoxin translocation, and microbial metabolic capacity. | Circulating microbial products and metabolite profiles may reflect HF-related congestion, hypoperfusion, barrier dysfunction, inflammation, and treatment effects. | Bioavailability is indirect and pathway-specific because dysbiosis represents a community state rather than a single metabolite exposure. | Renal and systemic disease burden may shape microbial metabolite accumulation and interpretation. | Clinical dysbiosis findings do not prove that a single microbial feature directly drives myocardial mitochondrial dysfunction. | Mostly indirect evidence through gut barrier, inflammatory, metabolic, and intervention-context findings; direct myocardial mitochondrial evidence is limited. | This row emphasizes reverse causality: HF may reshape the gut microbiome, so community-level signals should not be treated as direct mitochondrial mechanisms without causal validation. | (9, 10, 11, 45, 46, 67, 78) |

**Abbreviations:** AHR, aryl hydrocarbon receptor; ATP, adenosine triphosphate; CKD, chronic kidney disease; CYP1B1, cytochrome P450 family 1 subfamily B member 1; eGFR, estimated glomerular filtration rate; FMO, flavin-containing monooxygenase; HF, heart failure; HFpEF, heart failure with preserved ejection fraction; IPA, indole-3-propionic acid; PAGln, phenylacetylglutamine; SCFAs, short-chain fatty acids; SIRT3, sirtuin 3; TMA, trimethylamine; TMAO, trimethylamine N-oxide.
